# Brain stereotactic biopsy flow cytometry for central nervous system lymphoma characterization: advantages and pitfalls

**DOI:** 10.1186/s13046-016-0404-1

**Published:** 2016-08-27

**Authors:** Iole Cordone, Serena Masi, Mariantonia Carosi, Antonello Vidiri, Francesco Marchesi, Mirella Marino, Stefano Telera, Alessia Pasquale, Andrea Mengarelli, Laura Conti, Edoardo Pescarmona, Andrea Pace, Carmine M. Carapella

**Affiliations:** 1 Clinical Pathology, Regina Elena National Cancer Institute, Rome, Italy; 2 Pathology, Regina Elena National Cancer Institute, Rome, Italy; 3 Radiology, Regina Elena National Cancer Institute, Rome, Italy; 4 Hematology and Stem Cell Transplant, Regina Elena National Cancer Institute, Rome, Italy; 5 Neurosurgery, Regina Elena National Cancer Institute, Rome, Italy; 6 Neuroncology, Regina Elena National Cancer Institute, Rome, Italy

**Keywords:** PCNSL, Brain stereotactic biopsy, Flow cytometry, Tumor side population

## Abstract

**Background:**

Brain stereotactic biopsy (SB) followed by conventional histopathology and immunohistochemistry (IHC) is the gold standard approach for primary central nervous system lymphoma (PCNSL) diagnosis. Flow cytometry (FCM) characterization of fine-needle aspiration cytology and core needle biopsies are increasingly utilized to diagnose lymphomas however, no biological data have been published on FCM characterization of fresh single cell suspension from PCNSL SB. The aim of this study was to establish the feasibility and utility of FCM for the diagnosis and characterization of brain lymphomas from a tissue samples obtained by a single SB disaggregation.

**Methods:**

Twenty-nine patients with a magnetic resonance suggestive for PCNSL entered the study. A median of 6 SB were performed for each patient. A cell suspension generated from manual tissue disaggregation of a single, unfixed, brain SB, was characterized by FCM. The FCM versus standard approach was prospectively compared.

**Results:**

FCM and IHC showed an high degree of agreement (89 %) in brain lymphoma identification. By FCM, 16 out of 18 PCNSL were identified within 2 h from biopsy. All were of B cell type, with a heterogeneous CD20 mean fluorescence intensity (MFI), CD10 positive in 3 cases (19 %) with surface Ig light chain restriction documented in 11 cases (69 %). No false positive lymphomas cases were observed. Up to 38 % of the brain leukocyte population consisted of CD8 reactive T cells, in contrast with the CD4 positive lymphocytes of the peripheral blood samples (*P* < 0.001). By histopathology, 18 B-PCNSL, only one CD10 positive (5 %), 1 primitive neuroectodermal tumor (PNET) and 10 gliomas were diagnosed. A median of 6 days was required for IHC diagnosis.

**Conclusion:**

Complementary to histopathology FCM can contribute to a better characterization of PCNSL, although necrosis and previous steroid treatment can represent a pitfall of this approach. A single brain SB is a valid source for accurate FCM characterization of both lymphoma and reactive lymphocyte population, routinely applicable for antigen intensity quantification and consistently documenting an active mechanism of reactive CD8 T-lymphocytes migration in brain lymphomas. Moreover, FCM confirmed to be more sensitive than IHC for the identification of selected markers.

## Background

Primary central nervous system lymphoma (PCNSL), a rare hematological disease, represents a big challenge for clinicians and researchers [1, 2]. Despite improved therapeutic approaches, the diagnostic - prognostic characterization has documented little improvement and long-term response to treatment is rare [3, 4]. More than 90 % are diffuse large B-cell lymphomas (DLBCL) derived from a late germinal center B cell [5]. PCNSL is usually a widespread - diffusely infiltrating lymphoma. Thereafter, efforts at resection are discouraged, although resection may be associated with prolonged survival in surgically safe, single, PCNSL lesions [6]. Hence, stereotactic biopsy (SB) followed by conventional histopathology and immunohistochemistry (IHC) is the gold standard diagnostic approach useful to perform a differential diagnosis among other brain lesions, such us gliomas [7–11].

Flow cytometry (FCM) is a proven valuable diagnostic tool for the diagnosis and monitoring of hematological malignancies in routine clinical practice [12, 13]. More recently, FCM immunophenotype of fine-needle aspiration and/or core needle biopsy from lymphoid tissues has shown to improve diagnostic accuracy in B-cell non-Hodgkin lymphomas classification [14–18] and FCM significantly increases the sensitivity and specificity of cerebrospinal fluid (CSF) infiltration detection in onco-hematology [19–22]. However, no biological data have been published on FCM immunophenotype of fresh single-cell suspension obtained from SB of brain lymphomas.

We performed a feasibility study to evaluate the diagnostic and potential added value of FCM characterization of a single SB taken in vivo from intra-cerebral suspected lymphomas. FCM was compared with conventional IHC results in terms of diagnosis reliability and sensitivity in cell markers identification. The membrane CD20 mean fluorescence intensity (MFI) was evaluated to document, in PCNSL, the intensity of expression of a surface molecule potentially related to clinical response to Rituximab [23]. Finally, the tumor lymphoid side-population was characterized and compared to the peripheral blood (PB) lymphocytes phenotype, to investigate the blood-brain barrier permeability to non-malignant lymphocytes in CNS lymphomas.

## Methods

### Patients

Twenty-nine patients who underwent diagnostic SB for CNS tumor at Regina Elena National Cancer Institute were evaluated. All cases entered the study because of a magnetic resonance suggestive for PCNSL. About 50 % of enrolled patients underwent a previous steroid treatment to limit the mass-related cerebral edema before SB. The study was approved by our Institutional Ethics Committee and a signed informed consent was obtained from all the patients.

### Contrast-enhanced magnetic resonance

Magnetic Resonance Imaging (MRI) was performed on a 1.5-T system (Optima MR 450w, GE Health-care, Milwaukee, Wisconsin) with dedicated 16-channels receive-only RF coils; slice thickness 3 mm and matrix size of 512 × 512 were used. Before contrast medium infusion Spin-echo (SE) sequences T1, T2 and FLAIR in axial (T1, T2 e FLAIR) e cornal (T2) planes were performed. After contrast medium infusion (0.1 mmol/Kg body weight of gadopentetate dimeglumine contrast agent was administered intravenously, at a rate of 2 ml/s.) a volumetric contrast-enhanced T1-weighted GRE sequence was obtained (TR/TE = 7.8 ms/3.2 ms, matrix size = 512 × 512, voxel size = 0.5 x 0.5x 0.5 mm^3^) in axial plane with a reconstruction in coronal and sagittal planes to obtain very thin section with fat-suppressed images. Diffusion Imaging (DWIs) were obtained by single-shot SE EPI using two b value (0-1000).

**Fig. 1:**
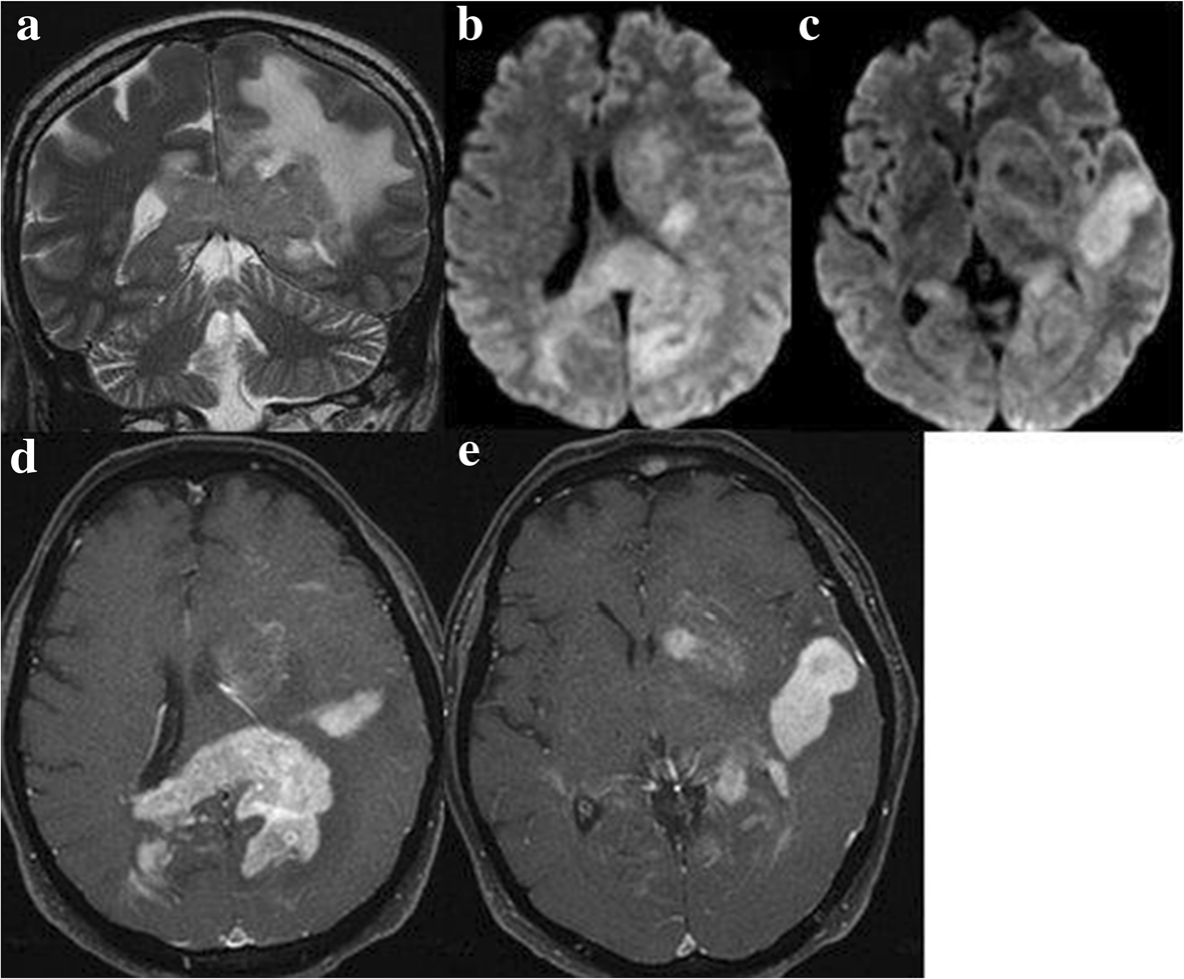
MRI findings strongly suggestive for brain lymphoma: SE T2 sequences in coronal plane (**a**), diffusion in axial plane (**b**, **c**) and fat-suppressed T1 after contrast medium infusion in axial plane (**d**, **e**). MRI shows multiple bilateral, periventricula sites with low signal intensity on T2, with oedema, hyperintensity signal on DWI and homogensously enhancement after contrast medium infusion

The MRI criteria for subject inclusion into the study were: 1) single or multiple lesions with periventricular sites; 2) low signal intensity on T2 weighted sequences; 3) homogeneously enhancement mass; 4) marked oedema; 5) hyperintense on DWI and hypointense on ADC maps; 6) enhancement of cranial nerve with increase nerve diameter [10] (Fig. 1). The MRI findings were evaluated by two neuroradiologists with 20 and 10 years of experience, respectively.

### Flow cytometry on brain stereotactic needle biopsy

Brain SB were performed using the Cosman-Roberts-Wells frame (Radionics, Burlington, Massachusetts). A median of 6 (range 5-8) tissue biopsies, with a medium size of 1 x 10 mm, were obtained. Immediately after extraction, each specimen was fixed with formalin for pathological evaluation and one biopsy was collected in a tube without any transport medium for FCM analysis.

The cell suspension was generated from a single, unfixed, SB fragment by gentle manual disaggregation in a sterile Petri dish containing 2-4 ml of phosphate buffered saline (PBS, Gibco by Life Technologies, UK) using a 10-mL syringe plunger rod. The released cells were collected and washed twice in PBS for 5 min at 1600 revolutions per minute. The supernatant was discarded and the pellet of cells was suspended in PBS and stained, according to the manufacturer’s recommendations, using a 6-colour panel of antibodies (Fitc/PE/PerCP/PE-Cy7/APC/APC-Cy7) and the “Duo-lyse” program of the Becton Dickinson Bioscience (BDB) Lyse-Wash-Assistant according to the following combinations: 1) CD3/CD56/CD45/CD4/CD19/CD8; 2) CD5/CD10/CD45/CD2/CD79b/CD20; 3) surface immunoglobulin (sIg) Ig lambda/sIg kappa/CD45/CD34/CD22/CD14, all from Becton Dickinson Bioscience (BDB). The FCM characterization was performed within 1 to 2 h from biopsy. The whole volume of sample was acquired and analyzed using a BDB FACSCanto flow cytometer and BDB theFACSDiva software.

The CD45 positive cells were gated and the percentage of B, T and myeloid population were evaluated. Surface Ig light chain expression was evaluated on the CD22 positive population; CD4 and CD8 expression was evaluated as the percentage of total CD3 positive lymphocytes. The markers expression was reported as percentage of positive cells within the CD45 positive population. The reactive population of small lymphocytes was utilized as positive/negative internal control. The MFI ratio for the CD20 antigen was calculated by comparison with negative control. An immunoperoxidase technique on fixed cytospins was utilized to investigate the presence of intra-cytoplasmic Ig light chains expression in the surface negative cases, as previously described [24].

Immunofenotype was also performed on CSF samples of 5 PCNSL cases. A volume of 3.5 mL (range 2.8-10) of CSF was collected in a tube without any transport medium and processed within a few (1-3) hours from collection to minimize cell loss.

### Flow cytometry on peripheral blood samples

The peripheral blood T/NK lymphocytes subset was evaluated by FCM according to the following combination: CD3/CD56/CD45/CD4/CD19/CD8. The lymphocytes phenotype was evaluated gating on the CD45 bright lymphoid cells. The CD4 and CD8 subset was evaluated on the percentage of total CD3 positive T lymphocytes.

### Histopathology

All tumors were classified according to the World Health Organization (WHO) classification [25] by conventional histology (H&E) and IHC on formalin-fixed, paraffin embedded SB tissue, utilizing the following antibodies: CD20, CD10 (clone 56C6), CD138, CD5, (Novocastra, Menarini, Florence, Italy), CD3, CD45 (CLA), CD79a, Ki-67, CD34 (QBEND clone), CD68 (KP1) p53 and GFAP (Dako Milan, Italy), CD56 (NCAM) (UCS Diagnostic, Rome, Italy) by a streptavidin-biotin enhanced immunoperoxidase technique, according to the manufacturer’s recommendations. The study was conducted in double-blind, with the hematopathologists and the cytometrists not aware of each other’s interpretation.

### Statistical analysis

Concordance analysis was performed to assess the degree of agreement (concordance) between FCM and IHC in PCNSL diagnosis. Fisher’s exact test was conducted to evaluate the different distribution between brain and PB lymphoid T sub-populations.

## Results

### Patients

Twenty-nine patients entered the study. The median age was 60 years (range 14–81), 16 patients were male and 13 female; all were HIV negative.

### Histopathology

Eighteen PCNSL, 1 primitive neuroectodermal tumor (PNET) and 10 gliomas were diagnosed by histopathology. By IHC, all PCNSL were CD45 CD20 CD79a positive, CD34 negative, one case was CD10 positive with a low intensity of expression, all with >70 % Ki-67 positive cells. A minority (5 to 10 %) of CD3 and CD5 reactive T lymphocytes scattered between the large lymphoma B cells was documented in 17 cases. By contrast, more than 30 % of the leucocytes was represented by a reactive perivascular T-cell infiltration (RPVI) in one case, as confirmed by FCM (Table 1, case n° 2). In 2 cases a diffuse necrosis with a small residual foci of lymphoma was documented by IHC. The PNET was CD56/CD34 positive CD45 negative, with a significant proportion of histiocytes and a minority of T lymphocytes among the tumor cells. The ten cases of gliomas were histologically classified as high-grade and low-grade in 8 and 2 cases, respectively. A median time of 6 days (5–8) was required between biopsy and diagnosis.

**Table 1:** Flow cytometry analysis of the CD45+ population in 16 PCNSL stereotactic biopsy shows a prevalence of large B cells sided by reactive T lymphocytes and monocytes

		N°	% Large B Lymphoma Cells					% Small reactive T lymphocytes	% Myeloid cells
Case n°	Histological type	CD45+ cells	CD19+	CD79b+	CD10+	CD19/Kappa + light chain	CD19/Lambda + light chain	CD3+	CD4dim or CD14+
1	DLBCL	42864	75	70	1	2	3	10	8
2	DLBCL	44073	60	65	0	93	2	38	1
3	DLBCL	91746	65	61	0	2	1	18	9
4	DLBCL	78828	80	76	70	85	0	8	3
5	DLBCL	66480	85	0	0	0	0	11	1
6	DLBCL	91726	95	92	0	2	96	5	0
7	DLBCL	113162	96	93	0	90	0	1	3
8	DLBCL	132550	96	91	0	0	83	1	3
9	DLBCL	38657	96	89	0	97	0	3	1
10	DLBCL	55841	70	68	0	96	0	24	1
11	DLBCL	27870	81	78	0	96	1	20	3
12	DLBCL	85000	95	92	0	0	0	5	4
13	DLBCL	61640	77	70	95	95	0	21	4
14	DLBCL	72001	88	12	0	0	0	10	2
15	DLBCL	30660	88	10	83	0	97	10	2
16	DLBCL	49523	72	26	0	0	98	26	2

*DLBCL* diffuse large B-cell lymphoma

### Brain stereotactic biopsy characterization by flow cytometry

#### PCNSL markers expression

**Fig. 2:**
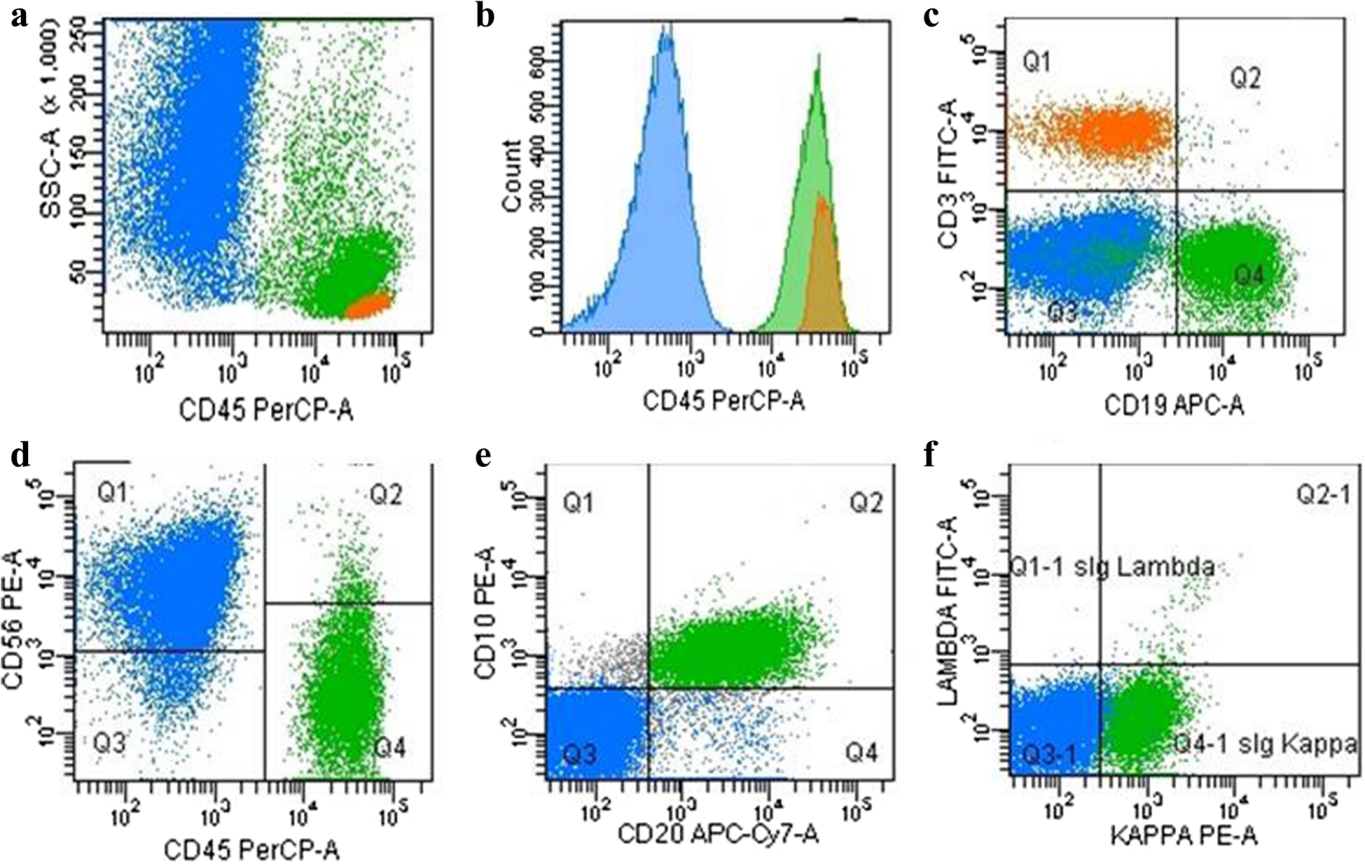
Flow cytometry analysis of cell suspension from one brain stereotactic needle biopsy of PCNSL. Blue color has been utilized to mark CD56 positive/CD45 negative brain cells (**a**, **b**, **d**), green for CD10 CD19 CD20 sIg-Kappa light chain positive large lymphoma cells (**c**, **e**, **f**), orange for the side population of CD3 positive T lymphocytes (**a**, **b**, **c**)

Sixteen out of 18 PCNSL (89 %) were identified by FCM. Leukocyte population was characterized within 2 h from SB, with a median of 66059 (range 27870–132550) CD45 positive cells and a proportion of 60 % to 96 % tumor B cells in all lymphomas (Table 1). Unequivocal identification of large lymphoma CD19 positive B cells (green color), CD3 small reactive T lymphocytes (orange color) and CD45 negative CD56 positive brain/neuro-ectodermal cells (blue color) was obtained in a single tube analysis (Fig. 2). All PCNSL were CD19/CD22 positive, CD20 positive in 15/16 cases (92 %), CD79b positive in 13/16 cases (81 %), CD5-CD34 negative, with a surface Ig light chain restriction detected in 11 cases (69 %), 7 Ig Kappa and 4 Ig Lambda light chain, respectively (Table 1). No intra-cytoplasmic light chain expression was documented by immunoperoxidase technique in the surface negative cases. Three cases were CD10 positive with surface Ig light chain restriction, consistent with the germinal center origin of the lymphoma. The CD20 expression was heterogeneous, with a median MFI ratio of 24.13 (range 7.55–58.55). One case was CD20 negative by FCM and weak positive by IHC. In 2 cases (11 %) FCM failed to identify a CNS lymphoma. Focusing on the 2 brain lymphomas not identified by FCM, a diffuse necrosis with a small residual foci of lymphoma was documented by IHC in both cases. In one case, 4 out of 8 SB were negative for PCNSL by IHC; in the second case, a prominent necrosis (>50 % of the available tissue) was documented in all 8 biopsies evaluated by histopatology. Both of failed cases occurred in patients who underwent a previous steroid treatment.

#### Tumor-side population phenotype

From 1 % to 38 % of the leukocyte population was represented by CD2/CD3/CD5 positive reactive T lymphocytes with a prevalence of CD8 positive cells documented in 13/16 PCNSL cases (81 %) and more than 40 % of CD56 positive T cells in two (Table 2, cases n°1 and n°14). A minority (<10 %) of CD4 dim CD14 positive monocytes among the lymphoma cells was also documented.

**Table 2:** Flow cytometry analysis of the lymphoid T reactive population from 16 PCNSL SB and corresponding peripheral blood samples

	McAb	% and CD4/CD8 ratio of reactive lymphocytes in SB and PB samples															
Case n°		1	2	3	4	5	6	7	8	9	10	11	12	13	14	15	16
Brain Streotactic Biopsy	CD3	84	86	91	85	89	98	98	98	95	94	95	98	85	97	87	85
	CD5	75	79	91	75	89	95	97	98	83	96	95	94	85	95	78	85
	CD56	60	2	7	4	4	1	1	5	3	6	1	1	2	40	1	1
	CD3/CD4	31	41	45	26	39	6	18	35	25	59	69	18	60	14	34	32
	CD3/CD8	87	57	50	59	47	72	60	42	74	28	16	71	33	56	53	45
	CD3/CD56	57	4	6	2	6	6	17	5	2	7	1	6	5	42	9	4
	T4/T8 ratio	0.35	0.72	0.9	0.44	0.83	0.08	0.3	0.83	0.34	2.1	4.3	0.25	1.8	0.25	0.64	0.71
Peripheral blood samples	CD19	nd	nd	nd	12	16	40	16	59	nd	29	19	15	nd	nd	11	9
	CD3	nd	nd	nd	64	63	48	55	16	nd	48	54	72	nd	nd	69	89
	CD56	nd	nd	nd	7	21	15	20	17	nd	23	25	20	nd	nd	20	11
	CD3/CD4	nd	nd	nd	73	59	50	79	66	nd	37	51	58	nd	nd	53	57
	CD3/CD8	nd	nd	nd	23	36	46	19	32	nd	62	47	38	nd	nd	43	38
	T4/T8	nd	nd	nd	3.17	1.6	1.08	4.15	2	nd	0.59	1.08	1.5	nd	nd	1.23	1.5

*nd* not done

#### PCNSL negative cases

A prevalence of CD45 negative, CD56 positive cells, consistent with a population of neuro-ectodermal origin, was identified in 13 cases, flanked by a minority of CD3 positive reactive T lymphocytes. In the PNET case, a prevalence (70 %) of CD45 negative, CD56/CD34 positive cells was documented flanked by a population of CD45/CD4dim/CD14 positive monocytes and CD4 reactive T lymphocytes.

Comparing FCM with IHC by concordance analysis, a statistically significant agreement (*P* = 0.0034) with a Cohen’s kappa coefficient of 0.859 was found.

### CSF flow cytometry of PCNSL cases

Lumbar puncture yielded adequate material for FCM analysis in all the cases analyzed. The CSF samples had a median cell count of 3 cell/μL (range 1-5). Despite the low CSF absolute cell number, a median of 6229 (range 1436-8285) evaluable cells were analyzed. The cell population was represented by a prevalence of CD4 positive T lymphocytes (76 %; range 65–93) in all the cases, sided by monocytes (CD14 positive = 6 %; range 1–13). In one case, despite the low cell count (1 cell/μL), 30 % of the lymphoid population was represented by clonal B cell, CD19 CD20 CD22 positive, CD5 CD10 negative, with surface Ig lambda light chain restriction (case n°6).

### Peripheral blood lymphoid asset

The peripheral blood T/NK lymphocytes subset was evaluated on 10/16 PCNSL. A lymphocytopenia, 900 cell/μL (range 350–1800), was documented. In contrast with the brain CD8 T lymphocytes prevalence (81 %), a population of CD4 positive T cells was documented in 9/10 (90 %) PB samples (*P* < 0.001) (Table 2).

## Discussion

This is a feasibility, prospective study that describes for the first time the use of FCM applied on a cohort of brain SB samples, providing evidence that a single SB is a valid source for identification and characterization of brain lymphomas and reactive infiltrating leucocytes, with a significant agreement between the FCM and IHC diagnosis (*P* = 0.0034). Despite the magnetic resonance strongly suggestive for PCNSL in 29 selected cases, 18 PCNSL, 10 gliomas and 1 PNET were diagnosed by IHC. FCM identified 16 out of 18 (89 %) PCNSL cases. Thereafter, 2 brain lymphomas (11 %) were missed by FCM. The overall experience indicates that FCM has difficulty detecting high-grade lymphomas, particularly DLBCL. Failure must be assigned to necrosis, cell fragility or inadequate sample size [15]. Moreover, necrosis can represent a major problem in PCNSL diagnosis after corticosteroid administration, as shown by a recently published study [26], considering that a large portion of newly diagnosed PCNSL patients often underwent a steroid treatment just before diagnosis, for clinical need. According to the study design, a single biopsy was analyzed by FCM while 6–8 samples were utilized for the IHC characterization. Nevertheless, the manual processing technique for tissue disaggregation allowed a successful FCM identification of brain lymphomas in 89 % of cases, confirming the limits of a correct diagnosis in patients pre-treated with steroids. Our study shows that a single SB is an adequate sample size for a comprehensive PCNSL characterization in absence of tissue necrosis. In fact, in the 2 PCNSL cases not identified by FCM the necrosis was the main factor. A diffuse necrosis with a small residual foci of lymphoma was documented by IHC in both cases, confirming the relevance of necrosis in hampering the diagnosis in large cell lymphomas. It is likely that performing FCM analysis on more than a single bioptic sample the rate of success in FCM brain lymphomas identification would increase.

FCM is a proven valuable diagnostic tool in hematological CSF infiltration detection [27, 28], increasing the sensitivity and specificity of leptomeningeal disease identification in CNS lymphomas [22]. We have recently documented that FCM can discriminate between reactive and neoplastic plasma cells in CSF samples with very low cell counts, confirming to be significantly more sensitive than standard approaches [21]. CSF FCM was performed on 5 out of 18 PCNSL cases and, despite the low cell count, a clonal B cell population was identified in one case. However, CSF cytology was negative and, although FCM was indicative of a B-cell clonal disorder, the data was not strong enough to reach the diagnosis of PCNSL. These findings are in agreement with previously published data [22].

A very wide panel of markers could be tested by FCM in a single brain SB, FCM demonstrate to be more sensitive than IHC for the identification of relevant markers. CD10 has been reported to be 10 times lower in PCNSL comparing to systemic diffuse large B-cell lymphomas [29]. In our study, the CD10 expression was documented in 3/16 PCNSL (19 %) by FCM and 1/18 (5 %) by IHC. The reason for this discordance is uncertain. Probably the different clones utilized for FCM (clone HI10a) and IHC (clone 56C6) could be involved in this matter. However, a different sensitivity between FCM and IHC in antigen identification is widely documented. Our report documents a possible underestimation of CD10 frequency of expression in PCNSL by IHC staining, highlighting the added value provided by performing FCM in terms of a more reliable technique for the identification of selected markers.

The panel of antigens used was in accordance with the WHO guidelines [25]. However, more recently, a significant differential expression of CD44 between Burkitt lymphoma and CD10 DLBCL has been documented, making CD44 an excellent candidate for rapid immunophenotypic discrimination between these two types of lymphoma [30]. Flanking IHC studies, FCM characterization, enriched by new relevant diagnostic markers, can significantly improve the diagnostic approach to PCNSL.

Surface Ig light chain restriction was documented in 11 cases. Immunoglobulin light chains restriction is an important adjunct to the use of surface markers in onco-hematology [13]. However, light chain restriction is not always documented on high grade B-cells NHL. In our series 5 cases were both surface and intra-cytoplasmic negative. Despite the lack of light chain restriction, the high FSC/SSC scatter of the B cell population (large cells) compared to the small T lymphocytes as well as the prevalence of B cells (85 %, range 60-96) compared to the T subset (10 %, range 1-38), strongly supported the diagnosis of a B cell neoplasm, confirmed by histopathology in all the cases. Moreover, since Ig light chain expression in non-Hodgkin lymphomas (NHL) is difficult to identify by IHC, cytometry can easily represent the method of choice for clonality detection in PCNSL.

The presence of a reactive immune response and perivascular T-cell infiltrate within the lymphoma has been shown to represent a prognostic biomarker and influence disease outcome in PCNSL [31, 32]. Antigen co-expression, mandatory for the classification of T and NK lymphocyte sub-populations, is easy to identity by FCM while difficult to assess by IHC. In this study, we documented a significant difference in lymphocyte sub-set distribution between brain lymphoma and peripheral blood. A prevalence of CD8 reactive T lymphocytes was documented in the majority (81 %) of PCNSL cases, while a prevalence of CD4 positive lymphocytes was identified in all but one peripheral blood samples (90 %) (*P* < 0.001). CNS is an immunogical sanctuary with restricted access and an unique microenvironment. The present study consistently shows that the blood-brain barrier actively selects a sub-population of CD8 non-malignant cells in PCNSL, providing a promising rationale for the investigation of cellular immunotherapy in brain tumors. To date, there is an increasing interest in engineered chimeric antigen receptor (CAR) T-cells in hematologic malignancies, also in NHL [33]. In this point of view, exploiting their migration to pathological lesions, reactive T-cells could be used for adoptive immunotherapy strategies based, for example, on targeting brain lymphoma CD19 positive B-cells by bi-specific T-cell engager monoclonal antibodies or engineered CAR T-cells in preclinical models.

Tumor associated macrophages, commonly present in NHL, have been related to poor prognosis and are potentially involved in the pathogenesis of brain NHL [34]. We identified a minority of CD4 dim CD14 positive monocytes among the B lymphoma cells and a great amount in the PNET case. Biological studies, aimed at a better understanding of the role of the tumor associated reactive population, would easily benefit from the FCM approach.

The identification of new therapeutic strategies is of prominent importance in PCNSL and FCM characterization can be a valuable tool for the detection of potential therapeutic targets and orientate tailored antibody-based therapies. The CD20 B cell-specific antigen is a membrane-bound protein whose expression is very heterogeneous among different B lymphoma subtypes [35] and significantly associated with disease prognosis [36, 37]. This difference may correlate with clinical response to Rituximab, a monoclonal antibody directed against the CD20 antigen that, in combination with conventional chemotherapy, has dramatically improved the survival of patients with diffuse large B-cell lymphoma [3]. By contrast, reduced CD20 expression has been associated with an inferior survival, suggesting that the evaluation of cell surface antigen intensity of expression can improve treatment tailoring of antibody-based therapy [38]. Anti-CD20 antibody therapy has shown to improve disease control and outcome in patients with primary CNS lymphoma [23, 39]. No biological data, however, is available regarding the intensity of surface antigen expression in intra-cranial B lymphomas. An objective quantification of antigen intensity of expression can be easily performed by FCM by the difference between antigen MFI and negative control. This technique is practical for use in routine setting. Utilizing this approach, our study documents for the first time a marked variability between surface CD20 expression level in brain lymphomas, ranging from very “bright” to “dim” in MFI with one CD20 negative case by FCM. CD20 (L26) staining is not usually graded by pathologists, and hematopathologists report all lymphomas that react with L26 antibody as “CD20 positive” regardless of expression level [40]. Therefore, the determination of CD20 MFI could represent a novel biomarker for a better stratification of PCNSL patients more likely to benefit from Rituximab treatment, for a more effective tailoring of the first line therapy. Actually, this hypothesis should be confirmed in further prospective studies. A prevalence of CD45 negative CD56 positive cells, consistent with a neuro-ectodermal population [41], was documented in 13 cases, with a strong CD34 expression identified in the PNET case, suggesting a potential role of FCM in non hematological tumors.

## Conclusions

This paper provides evidence that a single brain stereotactic core biopsy is a valid source for rapid identification and accurate FCM characterization of CNS lymphomas. FCM has shown to be more sensitive than IHC for selected markers, easily applicable for antigen intensity quantification and, documenting a prevalence of CD8 reactive T lymphocytes in brain lymphomas, suggests an active mechanism of lymphoid migration through the blood-brain barrier, potentially providing relevant information for further therapeutic decision making.

In the contest of the gold standard approach, the advantages of FCM over IHC include an excellent sensitivity, a short execution time, an objective quantification of antigen intensity of expression, a reliable approach for the evaluation of antigens co-expression on both tumor and tumor-side populations. The presence of necrosis remains a major disadvantage, as well as the requirement of fresh tissue for analysis. Finally, establishing a provisional diagnosis of brain lymphoma, FCM could positively affect the cost-benefit ratio, contributing in a shortening of time-to-treat interval with reduction of hospitalization indirect costs. Prospective studied will establish the synergic and complementary role of these two techniques for a better diagnostic approach and clinical management of brain lesions.

## Abbreviations

PCNSL: Primary central nervous system lymphoma
DLBCL: Diffuse Large B-Cell Lymphoma
SB: Stereotactic biopsy
IHC: Immunohistochemistry
CNS: Central Nervous System
FCM: Flow Cytometry
CSF: Cerebrospinal Fluid
MFI: Mean Fluorescence Intensity
PB: Peripheral Blood
MRI: Magnetic Resonance Imaging
SE: Spin-echo
DWIS: Diffusion Imaging
PBS: Phosphate Buffered Saline
BDB: Becton Dickinson Bioscience
WHO: World Health Organization
PNET: Primitive Neuroectodermal Tumor
RPVI: Reactive Perivascular T-cell Infiltration
NHL: Non-Hodgkin Lymphoma
CAR: Chimeric Antigen Receptor
